# Polyphenols in Oral Health: Homeostasis Maintenance, Disease Prevention, and Therapeutic Applications

**DOI:** 10.3390/nu15204384

**Published:** 2023-10-16

**Authors:** Yuanyuan Guo, Zhiquan Li, Feng Chen, Yujuan Chai

**Affiliations:** 1 School of Biomedical Engineering, Shenzhen University Medical School, Shenzhen 518060, China; 248gyy@163.com; 2 Key Laboratory of Optoelectronic Devices and Systems, College of Physics and Optoelectronic Engineering, Shenzhen University, Shenzhen 518060, China; 3 Guangdong Key Laboratory for Biomedical Measurements and Ultrasound Imaging, National-Regional Key Technology Engineering Laboratory for Medical Ultrasound, School of Biomedical Engineering, Shenzhen University Medical School, Shenzhen 518060, China; 4 Center for Healthy Aging, Department of Cellular and Molecular Medicine, University of Copenhagen, DK-2200 Copenhagen, Denmark; zhiquan@sund.ku.dk; 5 Central Laboratory, Peking University School and Hospital of Stomatology, Beijing 100081, China

**Keywords:** polyphenols, oral microbiota, functional food, oral caries, periodontal diseases, halitosis, oral cancer

## Abstract

Polyphenols, a class of bioactive compounds with phenolic structures, are abundant in human diets. They have gained attention in biomedical fields due to their beneficial properties, including antioxidant, antibacterial, and anti-inflammatory activities. Therefore, polyphenols can prevent multiple chronic or infectious diseases and may help in the prevention of oral diseases. Oral health is crucial to our well-being, and maintaining a healthy oral microbiome is essential for preventing various dental and systemic diseases. However, the mechanisms by which polyphenols modulate the oral microbiota and contribute to oral health are still not fully understood, and the application of polyphenol products lies in different stages. This review provides a comprehensive overview of the advancements in understanding polyphenols’ effects on oral health: dental caries, periodontal diseases, halitosis, and oral cancer. The mechanisms underlying the preventive and therapeutic effects of polyphenols derived from dietary sources are discussed, and new findings from animal models and clinical trials are included, highlighting the latest achievements. Given the great application potential of these natural compounds, novel approaches to dietary interventions and oral disease treatments may emerge. Moreover, investigating polyphenols combined with different materials presents promising opportunities for developing innovative therapeutic strategies in the treatment of oral diseases.

## 1. Introduction

Oral and periodontal diseases such as periodontitis are among the most common human diseases worldwide, leading to tooth loss and many other oral health complications [1]. As reported by the WHO, the population suffering from untreated dental caries, severe periodontitis, and oral diseases has reached 3.5 billion worldwide [2,3]. Oral health affects the quality of life significantly since poor oral health can affect one’s ability to eat, speak, and socialize comfortably [4]. Therefore, the prevalence of oral diseases has become a major global public health concern. During the past decade, intensive research has been conducted to prevent and treat oral diseases with nutrients, including identifying nutrients as dietary supplements and developing functional foods with health-promoting benefits.

Functional foods contain natural components that have been shown to prevent diseases and promote overall health. Modern diets such as ultra-processed foods and fruit juice beverages can reduce the influence of nutrients and their components in the oral cavity because they need to stay in the mouth for a longer period to exert their beneficial effects [5]. Many fruits, vegetables, and other food sources have been found to contain these beneficial components. Among these, polyphenols, which are a group of chemical compounds containing at least one phenol moiety, have been found to play a significant role in promoting human oral health and overall well-being [6,7,8]. These compounds have been extensively studied for their potential in preventing and treating dental issues.

Polyphenols are bioactive compounds that are naturally produced by plants, and essential in a wide range of plant functions. They contribute to the coloration of flowers, fruits, and seeds, which helps attract pollinators and seed dispersers [9]. Polyphenols also act as signal molecules that mediate plant-microbe interactions and help to defend against pathogens and predators. They are secondary metabolites that are commonly found in all human diets such as fruits, vegetables, and beverages such as tea and cocoa [10]. Polyphenols are well known for their potential health benefits for humans, including antioxidant, anti-allergic, anti-inflammatory, anticancer, antihypertensive, and antimicrobial effects [7,8,11]. They come into direct contact with oral tissues before being absorbed and metabolized, demonstrating great benefits for oral health directly and indirectly.

Due to the rapid accumulation of polyphenols’ functions studies in the field of oral health, several informative reviews have been published recently. The work of Flemming et al. provided a thorough introduction to the sources, and metabolic pathways of polyphenols, and their preventive effects in dentistry [10]. However, they did not mention the treatment application of these molecules. Kovac et al. reviewed the therapeutic potential of flavonoids and tannins, which are two major active compounds of polyphenols. The article focused on the origins of the molecules and their effects on the microorganism that causes oral infectious disease [9]. Some reviews emphasized the anticancer effect of polyphenols. For example, Trisha et al. discussed the benefits of tea polyphenols and covered a broad range of cancer types for their preventive functions [12], whereas Angellotti and colleagues selectively introduced the role of resveratrol in oral squamous cell carcinoma [13]. However, no review discusses the prevention and treatment effect of polyphenols in different types of oral disease and discusses the mechanism and progression of animal or clinical trials for the commercialization of functional food products.

In this review, progressions regarding the effects of polyphenols on four types of oral health, dental caries, periodontal diseases, halitosis, and oral cancer, were summarized. The mechanisms for the prevention and treatment effect of food-based polyphenols were listed, and the studies of oral disease in animal models as well as human patients were discussed, demonstrating recent achievements in the field. As these natural nutrients showed great commercial potential, new approaches to dietary interventions and oral disease treatments might be found. Safe and long-term oral health management based on polyphenol products of different formats might be beneficial to the overall well-being of humans.

### 1.1. Dietary Polyphenols

Dietary polyphenols refer to a class of natural compounds found in food that exhibit various biological activities and health benefits. Dietary polyphenols constitute a large group of compounds encompassing thousands of different substances. They are mainly categorized into flavonoids, phenolic acids, lignans, and stilbenes [14]. Each category includes numerous individual compounds that are present in various types of foods, such as fruits, vegetables, tea, red wine, and nuts (Table 1). Flavonoids are secondary metabolites derived from chalcones and fall into various subcategories, including flavanols, flavanones, flavonols, flavones, isoflavones, anthocyanins, proanthocyanidins, etc. [15]. Non-flavonoids contain phenolic acids (like caffeic acid), lignans, and stilbenes (e.g., resveratrol). Tannins can be further divided into hydrolyzable tannins and condensed tannins [16].

One of the primary functions of dietary polyphenols is their antimicrobial [17] and antioxidant activity, which contributes to overall health. They can neutralize free radicals, reduce oxidative damage to cells and tissues [18], and help prevent chronic diseases such as cardiovascular diseases [19], cancer [20], and inflammatory conditions [21]. Some polyphenols, such as flavonoids, are believed to be particularly beneficial for cardiovascular health. They can lower blood pressure, improve blood vessel function, and reduce the risk of heart disease and stroke [22]. Previous studies suggest that dietary polyphenols may have potential benefits in cancer prevention and treatment. They can inhibit the growth and migration of tumor cells while supporting the healthy functioning of the immune system [23]. These compounds also show anti-inflammatory effects and facilitate alleviating symptoms of inflammatory conditions [21]. Moreover, dietary polyphenols may have positive effects on blood sugar control, cognitive function, digestive health, and weight management, among other areas of health [24].

### 1.2. Oral Microbiota, Polyphenols, and Oral Health

Oral microbiota refers to the community of microorganisms that reside in the mouth. It is an essential component of the human microbiota and consists of hundreds to thousands of diverse species, including bacteria, fungi, viruses, and other microorganisms [25,26]. These microorganisms colonize different areas of the mouth, such as the teeth, gums, tongue, and saliva, forming a complex ecological community that influences oral and systemic health [25,27]. In the past century, medical research has primarily focused on studying bacteria in their planktonic phase. However, it is now widely recognized that oral microorganisms form biofilms [28,29]. Dental plaque, which forms on non-shedding surfaces in the oral cavity, meets all the criteria for a microbial biofilm and undergoes a process called succession [26]. When the delicate balance of the oral ecosystem is disrupted either due to an overload of microorganisms or a weakened immune system, a threat can be posed to local and systemic health.

The oral microbiota plays a crucial role in maintaining oral health by contributing to the digestion of food, protecting against harmful pathogens, and modulating the immune response [30]. Certain bacteria in the oral produce acids that help regulate the pH and prevent the growth of harmful bacteria that thrive in acidic environments [31]. Imbalances or dysbiosis in the oral microbiota can lead to oral diseases such as dental caries, periodontal disease, and oral infections. A significant alteration in the diversity and relative abundance of bacteria was found in the saliva of oral squamous cell carcinoma (OSCC) patients. These alterations can be used as biomarkers for the monitoring of the development, progression, and recurrence of oral cancer [32]. Moreover, increasing evidence supports that many systemic diseases are associated with disturbances in the oral ecosystem, such as diabetes, cardiovascular diseases, and tumors [33].

As the human diet undergoes major shifts throughout evolution, a significant change was identified in the composition of the oral microbiota. The diet provides nutritional resources for the oral microbiota and also acts as a selective pressure, favoring the growth of organisms that are best adapted to utilize specific dietary resources derived from the host [34]. Recent studies have been focused on exploring the potential of foods and diets in promoting oral health and preventing diseases. This has resulted in the development of foods and beverages that contain “functional ingredients”, which offer additional benefits for maintaining oral health or preventing the onset and progression of dental issues. In the process of research, more and more studies have found that polyphenols play a critical role in oral health. Research on the relationship between diet polyphenols and oral health is essential for promoting oral health by targeting oral bacteria and biofilms, understanding the impact of food processing on polyphenol effectiveness, and exploring the potential systemic health benefits of maintaining good oral health.

## 2. Method for Literature Search

**Figure 1:**
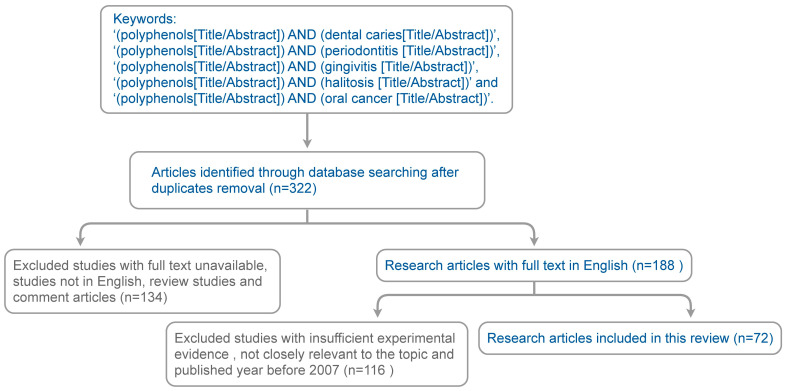
Flowchart of the search strategy and the literature selection process.

A comprehensive search of the literature was performed using PubMed, Web of Science, Embase, and Google Scholar. We focused on the articles examining the effects of polyphenols on various aspects of oral health, including dental caries, periodontal diseases, halitosis, and oral cancer. The keywords used for searching were: ‘(polyphenols[Title/Abstract]) AND (dental caries[Title/Abstract])’, ‘(polyphenols[Title/Abstract]) AND (periodontitis [Title/Abstract])’, ‘(polyphenols[Title/Abstract]) AND (gingivitis [Title/Abstract])’, ‘(polyphenols [Title/Abstract]) AND (halitosis [Title/Abstract])’ and ‘(polyphenols[Title/Abstract]) AND (oral cancer [Title/Abstract])’. After the removal of the duplicates, reviews, comments, non-English articles, and those without full text, 188 research papers were obtained. The quality of these studies was evaluated and studies with insufficient experimental evidence or not closely related to the scope of this review were excluded. Finally, a total of 72 studies were selected for this review (Figure 1).

## 3. Polyphenols and Oral Health

**Figure 2:**
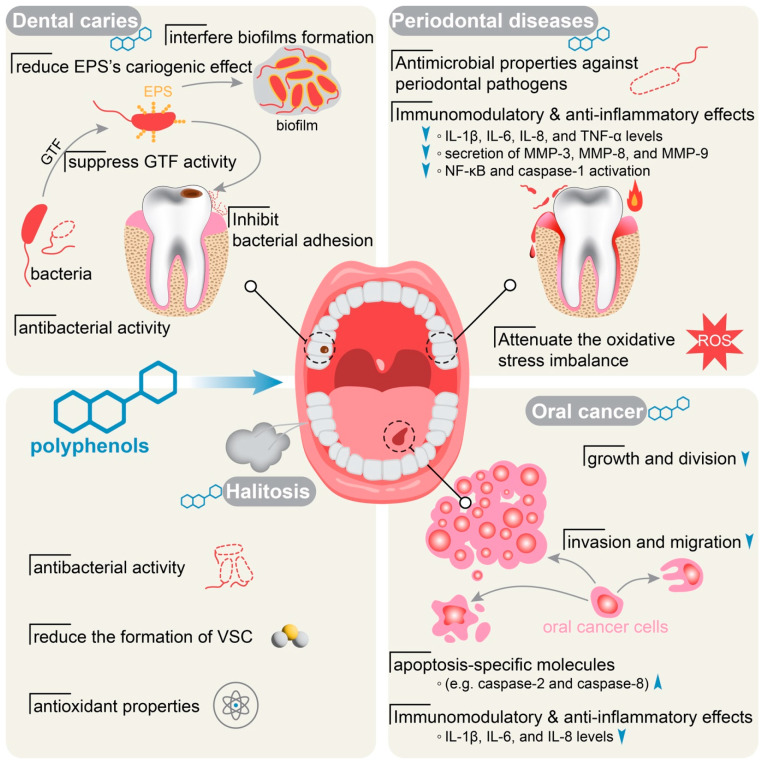
Polyphenols in dental caries, periodontal diseases, halitosis, and oral cancers. In dental caries, polyphenols have been investigated for their antibacterial properties, effectively suppressing bacterial growth and adhesion. They also inhibit glycosyltransferase enzyme (GTF) activity, reduce the cariogenic impact of exopolysaccharides (EPS), and disrupt biofilm formation. In conditions involving inflammation, bleeding, and gum recession, polyphenols offer antimicrobial effects, enhance immunomodulation, and exhibit anti-inflammatory properties. They help mitigate oxidative stress, a critical factor in periodontal diseases. For halitosis, polyphenols possess antibacterial and antioxidant properties. In the meantime, they help reduce the volatile sulfur compounds (VSC), the primary source of halitosis. Regarding oral cancers, polyphenols have a multifaceted impact on oral cancer cells, including the inhibition of growth and division, decreased invasion and migration, enhanced apoptotic activity, and reduced expression of inflammatory cytokines such as IL-1β, IL-6, and IL-8. The small blue arrows indicate the upregulation/promotion or downregulation/inhibition of the biomarkers or behaviors.

Oral health is essential for preventing dental problems, maintaining overall health, and ensuring a good quality of life. To provide a comprehensive understanding of the impact of polyphenols on oral health, we will delve into the effects of these compounds on four aspects, including dental caries, periodontal diseases, halitosis, and oral cancer (Figure 2).

### 3.1. Polyphenols and Dental Caries

Dental caries, also known as tooth decay or cavities, is a chronic oral disease with high prevalence among children and adolescents. It’s a bacterial infectious disease that affects the hard tissues of the teeth and is caused by the interaction between bacteria, sugars, and carbohydrates in the mouth [35]. Dental caries is caused by a microecological imbalance in the biofilm of dental caries pathogens, with *Streptococcus mutans* (*S. mutans*) particularly involved [29,36]. *S. mutans* is a Gram-positive bacterium and is recognized as the most important cariogenic bacteria in the oral cavity, secreting exopolysaccharides (EPS) to form a three-dimensional biofilm structure [37]. Dental plaque biofilm appears where the bacteria attach and aggregate, protecting the bacteria and making them highly resistant to drugs and host immune defenses [38].

It is widely recognized that the consumption of dietary sugars, especially sucrose, is directly linked to the development of dental caries. The cariogenic bacteria metabolize sugars in the food and rapidly produce a large amount of organic acids, leading to microecological dysbiosis [39]. The acid production resulting from the metabolism of sugars in the biofilm leads to a decrease in environmental pH, which is responsible for the demineralization of hard dental tissues [40]. The bacteria produce acids that erode the enamel and dentin of the teeth, leading to the formation of cavities Therefore, effective methods to prevent the development of dental caries involve inhibiting the growth of pathogenic bacteria in the mouth, reducing the formation of EPS, and decreasing the content of biofilm.

Numerous studies have shown that polyphenols exhibit antibacterial activity against the *S. mutans* (Figure 2, Table 2) [8,41,42,43]. Babaeekhou and Ghane demonstrated that extracts of ginger have high antibacterial activity against *S. mutans* and *S. sobrinus* [44]. Ethanolic extract of Polish propolis inhibits the growth of *S. mutans* and demonstrated the MIC value for tested strains at the range of 25–50 µg/mL [41]. Polyphenols also inhibit the growth and activity of other cariogenic bacteria such as *Lactobacillus rhamnosus* (*L. rhamnosus*), *Candida albicans* (*C. albicans*), and *Fusobacterium nucleatum* (*F. nucleatum*) (Table 2) [45,46,47].

As reported above, biofilms play a crucial role in the survival of *S. mutans.* Polyphenolic compounds can also interfere with the formation of biofilms, and protect the tooth from bacteria adherence [36,46,48,49]. Catechins identified from hot steeping green or black tea showed inhibitory effects on biofilm formation and cell viability of *S. mutans.* and *S. sobrinus* [50]. It was demonstrated that tart cherry (*Prunus cerasus* L.) fractions had a dose-dependent inhibitory effect on biofilm formation. Additionally, they also reduced the adherence of *C. albicans* and *S. mutans* to a hydroxylapatite surface as well as the adherence of *F. nucleatum* to oral epithelial cells [46].

The EPS-rich matrix plays a crucial role in bacterial adhesion to tooth surfaces and provides mechanical stability for acidogenic and aciduric bacteria [29,38]. Therefore, EPS formation is essential for the pathogenesis of dental caries. Polyphenols have been reported to reduce the cariogenic effect of EPS in biofilm (Figure 2, Table 2) [47,51]. Recently, a study showed that *Lonicera caerulea* fruit polyphenols (LCP) affect the characteristics of EPS produced by *L. rhamnosus*. Specifically, the addition of LCP increases galactose in EPS and disrupts the original aggregation state of EPS [45]. However, LCP does not significantly affect the molecular weight and functional group composition of EPS. This suggests that LCP can alter the surface morphology, content, and composition of EPS produced by *L. rhamnosus* [45]. These changes in EPS may contribute to reducing the cariogenic effect of EPS and biofilm formation. Schneider-Rayman et al. found that epigallocatechin-3-gallate (EGCG) exhibited the antibacterial and antibiofilm activity of *S. mutans* in a dose-dependent manner [52]. At concentrations of 2.2–4.4 mg/mL, significant reductions were observed in *S. mutans* biofilm formation, DNA content, and EPS production. Furthermore, EGCG reduced the expression of genes involved in EPS production (gtfB, gtfC, and ftf) as well as genes involved in protection against oxidative stress (nox and sodA) [52].

In addition, polyphenols could inhibit the formation of glucan and biofilm from *S. mutans* by suppressing the activity of glycosyltransferase enzymes (GTF) (Figure 2, Table 2) [53,54,55]. Goto et al. illustrated that Roasted Green Tea (RGT)-specific polyphenols had GTF inhibitory activity and a strong inhibitory effect on biofilm formed by *S. mutans* [53]. A study in rats found that cranberry Proanthocyanidins (PAC) showed significantly less caries severity on smooth surfaces and sulcal surfaces. Furthermore, A-type PAC oligomers have shown effective inhibition of insoluble glucan synthesis by GtfB on saliva-coated apatitic surfaces. These PAC oligomers also had an impact on bacterial glycolysis [56]. Additionally, polyphenols may inhibit *S. mutans*-mediated acidification. It reduces the glycolytic pH drop and lactate production thus protecting from dental caries [57].

It is worth mentioning that the following investigations report that polyphenols can prevent the development of dental caries in humans. An in vivo study was performed using saliva and dental biofilm samples collected from 75 healthy subjects. The use of polyphenolic mouthwash resulted in a significant reduction in bacterial taxa associated with oral diseases in both the refined sugar group and the unrefined sugar group [58]. A one-year prospective human intervention study used fermented lingonberry juice (FLJ) as a mouthwash for 6 months, followed by a 6-month washout period. FLJ efficiently reduced visible plaques, *S. mutans*, and *Candida* levels, as well as caries risk [59]. A randomized blinded clinical study with 60 healthy children of age 9–14 found that 0.5% *Camellia sinensis* extract exhibited antiplaque activity over the 2-week experiment period. In the treatment group, the salivary pH increase was sustained and significant, and oral hygiene was well improved [60]. However, conventional anti-caries agents often exhibit limitations such as poor stability, low efficacy, or short residence time in the oral environment. It is critical to develop novel long-term strategies for the prevention of dental caries, especially for children and teenagers.

**Table 2:** Effects of polyphenols on dental caries.

Study Group	Active Components	Study Design	Pathogens	Cells/Tissues/Animals	Results
Ren. et al., 2023 [45]	*Lonicera caerulea* fruit polyphenols	In vitro	*L. rhamnosus* (RYX-01)	N/A	Inhibition of RYX-01 growth Reduction of EPS and biofilm formation Inhibition of quorum sensing and biofilm formation-related gene expression
Pärnänen et al., 2023 [59]	Fermented Lingonberry Juice	A One Year Prospective Human Intervention Study (25 patients)	*S. mutans* *Candida* *Lactobacilli*	N/A	Reduction of *S. mutans* and *Candida* counts Increased *Lactobacilli* counts significantly Reduction in decayed surfaces (DS) index, bleeding on probing (BOP), and visible plaque index (VPI) No effect on probing pocket depths(DDPs)
Goto et al., 2023 [53]	Roasted Green Tea (RGT)-specific polyphenols	In vitro	*S. mutans*	N/A	Inhibition of *S. mutans* biofilm formation and GTF activity
Chhaliyil et al., 2022 [58]	Polyphenolic mouthwash	in-vivo study was performed using saliva and dental biofilm samples collected from 75 healthy subjects.	N/A	N/A	Reduction in bacterial taxa associated with oral diseases in refined sugar group and unrefined sugar group
Nomura et al., 2021 [43]	Flavedo, albedo, fruits, and leaves of *Citrus unshiu* extracts	In vitro	*S. mutans*	N/A	Inhibition of *S. mutans*
Yabuta et al., 2021 [57]	*Backhousia citriodora* (lemon myrtle) extract	In vitro	*S. mutans*	N/A	Reduction of the glycolytic pH drop Inhibition of lactate production No effect on lactate dehydrogenase activity
Xu et al., 2021 [61]	EGCG–phospholipid complex	In vitro	*S. mutans*	N/A	Strong antibacterial activity on *S.mutans* Reduction of acid production and tooth surface adhesion Inhibition of glucan and biofilm formation by suppressing the GTF activity
Schneider-Rayman et al., 2021 [52]	Green tea polyphenol, epigallocatechin gallate (EGCG)	In vitro	*S. mutans*	N/A	Inhibition of the planktonic growth and the biofilm formation Reduction of *S. mutans* EPS production Reduction in gtfB, gtfC, and ftf genes involved in EPS production, and the nox and sodA genes involved in the protection against oxidative stress
Magacz et al., 2021 [42]	Acetone extracts of *Reynoutria. japonica*, *R. sachalinensis*, and *R. x bohemica*	In vitro	*S. mutans*	N/A	Modulated the activity of the lactoperoxidase system
Goyal et al., 2021 [54]	Polyphenols gallic acid and tannic acid	In vitro	*S. mutans*	N/A	Inhibition of dextransucrase activity
Babaeekhou et al., 2021 [44]	N-hexane, ethyl acetate, methanol, and aqueous extracts of Ginger	In vitro	*S. mutans* *S. sobrinus*	N/A	Inhibition of *S. mutans* and *S. sobrinus*
Selvaraj et al., 2020 [62]	Toothpaste containing probiotics and Neem	In vivo (60 patients)	*S. mutans*	N/A	Reduction of bacterial count
Kim et al., 2020 [50]	Green or black tea extracts	In vitro	*S. mutans* *S. sobrinus*	N/A	Inhibition of biofilm formation, cell viability, and GTF activity Maintained the pH
Ben Lagha et al., 2020 [46]	Tart cherry (*Prunus cerasus* L.) extract	In vitro	*C. albicans* *S. mutans* *F. nucleatum*	Oral epithelial cell line GMSM-K, human oral epithelial cell line B11	Inhibition of biofilm formation Attenuated the adherence of *C. albicans* and *S. mutans* to a hydroxylapatite surface as well as the adherence of *F. nucleatum* to oral epithelial cells.
Veloz et al., 2019 [36]	Polyphenolic compounds in Chilean Propolis	In vitro	*S. mutans*	N/A	Inhibition of bacterial growth and biofilm formation
Philip et al., 2019 [51]	Extracts of cranberry, blueberry, and strawberry, and a combination of the three berry extracts (Orophenol)	In vitro	*S. mutans*	N/A	Reduction in biofilm metabolic activity, acid production, and EPS biovolumes No bactericidal on *S. mutans*
Farkash et al., 2019 [47]	Padma hepaten and a polyphenol extraction from green tea	In vitro	*S. mutans* *C. albicans*	N/A	Inhibition of biofilm formation without affecting the planktonic growth Reduction in EPS secretion
Yabuta et al., 2018 [48]	Extract from Lemon myrtle (*Backhousia citriodora*)	In vitro	*S. mutans*	N/A	Inhibition of *S. mutans* biofilm
Damiano et al., 2017 [49]	Ziziphus jujuba Mill fresh leaves	In vitro	*S. mutans*	N/A	Inhibition of biofilm bioactivity
Hambire et al., 2015 [60]	0.5% *Camellia sinensis* extract	A randomized blinded controlled trial with 60 healthy children of age 9–14 years	N/A	N/A	More effective compared to 0.05% sodium fluoride and 0.2% chlorhexidine gluconate mouth rinses
Koo et al., 2010 [56]	Cranberry PAC fraction	In vivo	*S. mutans*	Sprague-Dawley rats	Reduction of biofilm formation and smooth-surface caries Diminished the synthesis of insoluble glucans by GtfB adsorbed on a saliva-coated hydroxyapatite surface

### 3.2. Polyphenols and Periodontal Diseases

Periodontal disease, including gingivitis and periodontitis, is a chronic inflammatory condition that affects the tissues surrounding and supporting the teeth, including the periodontal ligament, connective tissue, and alveolar bone [63]. It is caused by the accumulation of dental plaque, a sticky film of bacteria, on the teeth and gingiva. Gingivitis is the early stage of periodontal disease, which is characterized by red, swollen, and bleeding gingiva [64]. Untreated gingivitis can progress to periodontitis, a more severe form of gingival disease.

Periodontitis is a complex disease that involves ongoing interactions between bacteria and the immune and inflammatory responses of the host, which are influenced by genetic and environmental factors. The homeostasis or symbiosis of oral microbiota plays a crucial role in periodontal health, which is characterized by a state of dynamic equilibrium between periodontal microflora and a controllable immune/inflammatory response of the host [65]. Dysregulation of the inflammatory response or stimulation of an imbalanced microbial community can disrupt the homeostasis in periodontal tissues. This dysbiosis results in an overgrowth of pathogenic bacteria and a decrease in beneficial bacteria, leading to a dysregulated and hyperinflammatory immune response [66]. Pathogenic bacteria such as *Porphyromonas gingivalis* (*P. gingivalis*), are reported to be associated with periodontal diseases. These bacteria produce virulent factors such as gingipains and trigger the activation of host-derived proteolytic enzymes such as matrix metalloproteinases (MMPs) [67]. The hyperinflammatory response is accompanied by the release of interleukin-1 (IL-1) superfamily members and the degradation of the extracellular matrix, which leads to chronic inflammation in the gingiva and the loss of alveolar bone [67,68].

Treatment for periodontal disease typically involves professional dental cleaning techniques to remove plaques and calculuses, scaling and root planing to remove bacteria from the periodontal pockets, as well as minimally invasive nonsurgical therapy (MINST) [65,68]. In advanced cases, surgical procedures may be necessary to repair damaged tissues and restore oral health. Traditional treatments such as mechanical debridement and antimicrobial strategies suffer from limitations and adverse effects. Thus, there is a growing interest in exploring alternative adjunctive therapies for the prevention and treatment of periodontal disease.

Studies have shown that polyphenols possess antimicrobial properties against periodontal pathogens, including *P. gingivalis*, *Aggregatibacter actinomycetemcomitans* (*A. actinomycetemcomitans*), *Staphylococcus aureus* (*S. aureus*), *Streptococcus mitis* (*S. mitis*) and *Fusobacterium nucleatum* (*F. nucleatum*) (Figure 2, Table 3) [69,70,71,72,73,74]. In vitro studies have demonstrated the inhibition of bacterial growth, adhesion to oral cells, and enzymatic activity of polyphenols such as chlorogenic acid [75], prenylated flavonoids [76], theaflavins [77], baicalein [78,79] and proanthocyanidins [70]. These polyphenols contribute to reducing the colonization and virulence of pathogens in the oral cavity, which is crucial for the prevention and treatment of periodontal disease.

In addition to their antimicrobial properties, polyphenols are also crucial in balancing oxidative stress and antioxidant activity in the oral cavity, which prevents the deterioration of periodontal tissue (Figure 2, Table 3) [80,81,82]. Polyphenols derived from berries (Brand name: Orophenol^®^) showed inhibitory effects on the growth of *P. gingivalis* and reduced its hemolytic activity. Additionally, Orophenol^®^ suppressed the production of reactive oxygen species (ROS) by oral keratinocytes stimulated with *P. gingivalis*. and exhibited a protective effect against damages caused by *P. gingivalis* [81]. A polyphenolic mixture extracted from the pomace of the Croatina grape variety also demonstrated antioxidant properties, downregulating inflammation in macrophages [80]. A study focused on human gingival fibroblasts (HGFs) revealed that treatment with a polyphenolic fraction of *L. caerulea* berries (10–50 μg/mL) resulted in a decrease in ROS production, intracellular glutathione (GSH) depletion, and lipid peroxidation in lipopolysaccharide (LPS)-treated cells [82].

Polyphenolic components exhibit immunomodulatory and anti-inflammatory effects in periodontitis as well (Table 3). They modulate the host immune response, leading to a decrease in pro-inflammatory cytokines and an increase in anti-inflammatory cytokines [6,83,84]. Several inflammatory mediators such as interleukin-8 (IL-8), IL-6, tumor necrosis factor α (TNF-α), nuclear factor Kappa-B(NF-κB), and histamine are involved in this process along with the LPS produced by periodontal pathogens [85,86]. PAC are rich in cranberry, wild berry, (*Vaccinium angustifolium* Ait.), blueberry, grape apples, cocoa, and green tea [71,87]. Studies have reported that PAC can suppress inflammation by reducing the level of IL-1β, IL-6, IL-8, and TNF-α through the downregulation of the MAPK/AP-1 signaling pathway, and the inhibition of the transcription function of NF-κB. Similarly, the secretion of MMP-3, MMP-8, and MMP-9 was also inhibited in a dose-dependent manner [87,88,89,90,91]. The research of Farzanegan et al. reported that the combination of resveratrol and silymarin effectively reduced the inflammatory effects of histamine on cultured HGFs through a decrease in the levels of IL-6, IL-8, TPA-1, and TNF-α [84]. Ben Lagha and Grenier showed that the aflavins, and black or green tea extracts all inhibited the activation of NF-κB and caspase-1 as well as the release of IL-1β by monocytes/macrophages. Tea polyphenols have anti-inflammatory properties and help reduce gingival epithelial barrier dysfunction caused by TNF-α [83]. Apple and hop bract polyphenols also inhibit *P. gingivalis*-mediated pMMP-9 activation and OSCC cellular invasion. Furthermore, polyphenols reduced the activation of heat shock protein 27 and Ets1 and NF-κB [92]. The administration of resveratrol-quercetin reduced the inflammatory process in apical periodontitis, periapical bone resorption, and altered the expression of osteoprotegerin, IL-10, and tartrate-resistant acid phosphatase in rats [93]. These findings indicate that the modulation of the immune response helps alleviate inflammation and promote tissue healing in periodontal disease.

Several clinical studies have investigated the effects of polyphenols on periodontal health (Table 3). These studies reported higher attachment levels, reduced inflammation, and decreased bone loss when treated with polyphenolic compounds. It is suggested that the specially formulated FLJ mouthwash may help relieve mild inflammation and proteolytic burden in the oral mucosa and periodontal tissues [59]. A randomized clinical study in patients with periodontitis (stage III–IV) showed that combined use of PAC in MINST resulted in better clinical outcomes for moderate pockets. Moreover, MMP-3 concentration in saliva was increased when PAC was incorporated, compared with MINST alone. These results indicated that MMP-3 concentration in saliva can be used as a biomarker for the assessment of periodontal health [94]. Additionally, a prospective, double-blind, randomized clinical trial conducted by Sánchez et al. also identified that oligomeric PAC can affect the condition of periodontal tissue [95]. Despite the promising clinical findings, the heterogeneity of studies makes it difficult to determine the clinical applicability of polyphenols in periodontal disease treatment.

Overall, the ability of polyphenols to inhibit bacterial growth, reduce inflammation, and promote tissue healing makes them promising materials for the prevention and adjunctive therapies of periodontal disease (Figure 2). However, further research is needed to standardize the dosage, delivery methods, and formulations of polyphenols to optimize clinical outcomes. The exploration of polyphenols provides new options for therapeutic approaches in the management of periodontal disease.

**Table 3:** Effects of polyphenols on periodontal disease.

Study Group	Plants/Active Components	Study Design	Pathogens	Cells/Tissues/Animals	Results
Ullah et al., 2023 [69]	*Cistus* × *incanus* L., *Scutellaria lateriflora* L.	In vitro	*P. gingivalis*	Human keratinocyte epithelial cells HaCaT	Inhibition of *P. gingivalis* growth Reduction in *P. gingivalis* HaCaT invasiveness and biofilm
Pärnänen et al., 2023 [59]	Fermented lingonberry juice (FLJ)	One-year prospective clinical intervention study (25 patients)	*S. mutans* *Candida* *Lactobacilli*	N/A	Reduction of *S. mutans* and *Candida* counts Increased *Lactobacilli* counts Reduction in decayed surfaces (DS) index, bleeding on probing (BOP), and visible plaque index (VPI) No effect on probing pocket depths(DDPs)
Alkimavičienė et al., 2023 [94]	Proanthocyanidins (PACs)	Clinical study in 46 patients with periodontitis	N/A	N/A	Inhibition of *S. mutans* biofilm formation and GTF activity Better clinical outcomes for moderate pockets Improved MMP-3 concentration in saliva
Vaillancourt et al., 2022 [81]	A berry polyphenolic fraction (Orophenol^®^) composed of extracts from cranberry, wild blueberry, and strawberry	In vitro	*P. gingivalis*	Human oral keratinocyte cell line B11	Inhibition of *P. gingivalis* growth Decreased *P. gingivalis* hemolytic activity, its adherence to a basement membrane matrix model, and its proteinase activities Reduction in production of ROS by oral keratinocytes stimulated with *P. gingivalis*
Qi et al., 2022 [96]	Turkish Gall’s effective constituent was prepared into nanoparticles (T-NPs) by the principle of oxidative self-polymerization.	In vitro	*P. gingivalis*	N/A	Stronger antibacterial activity on oral pathogens T-NPs induced bacteria lysis by promoting the excessive production of ROS without periodontal tissue damage
He et al., 2022 [97]	Tea polyphenols (TP) and AdipoRon (APR)	In vitro and in vivo	N/A	Bone marrow stromal cells BMSCs and RAW 264.7 cells Sixty 8-week-old male C57BL/6 mice	Programmed core-shell nanofibers for sequential and controlled release of tea polyphenols and AdipoRon Reduction of proinflammatory cytokines levels in vitro Promoted osteogenic differentiation in an inflammatory microenvironment in vitro Alleviated periodontal tissue inflammation and enhanced the regeneration of alveolar bone in vivo
Iviglia et al., 2021 [80]	A polyphenolic mixture extracted from the pomace of the Croatian grape variety	In vitro	N/A	Human osteoblast-like SAOS2 cells	Anti-inflammatory and antioxidant properties Reduction of acid production and tooth surface adhesion
Dal-Fabbro et al., 2021 [93]	Red wine consumption or its polyphenols	In vivo	N/A	3-month Wistar rats with apical periodontitis	Reduction of the inflammatory process in apical periodontitis and periapical bone resorption
Torre et al., 2020 [98]	Polyphenol-rich grape pomace extracts	In vitro	N/A	Human bone marrow stromal cells hMSC	Decreased receptor activator of nuclear factor κ-Β ligand Enhanced expression of genes involved in osteoblast differentiation
Galarraga-Vinueza ME, et al., 2020 [87]	Cranberry concentrates at 25, 50, and 100 µg/mL	In vitro	N/A	THP-1 cells (monocytic line, Human gingival fibroblasts (HFIB-G cell line) osteosarcoma-derived osteoblasts SAOS-2 cell line	Downregulated the expression of IL-8 and IL-6 in LPS-stimulated macrophages with cranberry concentrates at 50 and 100 µg/mL Upregulated the expression of IL-10 in LPS-stimulated macrophages by cranberry concentrates at 100 µg/mL
Ben Lagha, et al., 2020 [70]	Highbush blueberry proanthocyanidins	In vitro	*P. gingivalis*	Gingival keratinocyte cell line B11 In vitro gingival keratinocyte barrier model	Reduction in bacterial growth
Tsou et al., 2019 [75]	Coffee extract and its primary phenolic acid, chlorogenic acid	In vitro	*P. gingivalis*	N/A	Inhibition of *P. gingivalis* viability Reduction of associated protease activity.
Ben Lagha et al., 2019 [83]	Green and black tea extracts in distilled water 10 mg/mL EGCG, theaflavin fraction in 95% ethanol	In vitro	N/A	U937 human monocytes, human monoblastic leukemia cell line U937-3xκ B-LUC, gingival keratinocyte cell line B11	Inhibited the activation of NF-κB and caspase-1 as well as IL-1β secretion by monocytes/macrophages Protected keratinocytes against the TNF-α-mediated breakdown of barrier integrity.
Jekabsone et al., 2019 [71]	Pelargonium sidoides DC root extract (PSRE), proanthocyanidin fraction from PSRE (PAC)	In vitro	*P. gingivalis*, *S. salivarius*, *S. aureus*, *S. epidermidis*, *A. actinomycetemcomitans* and *E. coli.*	Human primary gingival fibroblasts HGF, Human peripheral blood mononuclear cells PBMCs	Strong antibacterial, anti-inflammatory, and gingival tissue-protecting properties under periodontitis-mimicking conditions
Farzanegan et al., 2019 [84]	Silymarin or resveratrol (100 μg/mL) and a combination of these two polyphenols	In vitro	N/A	Human gingival fibroblast cell line HGF-3	Inhibited inflammatory effects of histamine on cultured HGFs by reduction of IL-6, IL-8, TPA-1, and TNF-α
Ben Lagha, et al., 2019 [99]	Cranberry Proanthocyanidins (PAC)	In vitro	*A. actinomycetemcomitans*	U937 human monocytes	Reduction of leukotoxin (LtxA) gene expression Neutralized the cytolytic and pro-inflammatory responses of human macrophages
Khalil et al., 2019 [72]	Methanolic extract of *Salvadora persica*	In vitro	*S. aureus* and *Streptococcus sp.*	N/A	Inhibition of bacterial growth
Kariu et al., 2017 [76]	Prenylated flavonoids isolated from *Epimedium* species plant	In vitro	*P. gingivalis*	N/A	Inhibition of gingipains activity in a non-competitive manner Inhibition of *P. gingivalis* growth and biofilm formation
Díaz Sánchez et al., 2017 [95]	New nutritional supplement made of oligomeric proanthocyanidins (PAC)	A prospective, double-blind, randomized, controlled clinical trial in 20 patients	N/A	N/A	Oligomeric PAC affects periodontal tissue health but has no effect on the accumulation of plaque on the tooth surface
Ben Lagha et al., 2017 [100]	EGCG from green tea and theaflavins from black tea	In vitro	*F. nucleatum*	N/A	Inhibited the bacterial adhesion and *F. nucleatum*-induced hemolysis No effects on bacterial growth at antiadhesive concentrations
Ben Lagha et al., 2017 [101]	Theaflavins from black tea	In vitro	*P. gingivalis*	U937-3xκB-LUC monocyte cell line	Inhibition of Arg- and Lys-gingipain and bacterial adhesion Enhanced tight junction integrity of gingival keratinocytes
Tipton et al., 2016 [88]	Cranberry high molecular weight non-dialyzable material (NDM)	In vivo	N/A	Normal human gingival fibroblasts from a healthy patient with noninflamed gingiva	Inhibition of IL-6 and MMP-3 production by human gingival fibroblasts
Inaba et al., 2016 [92]	Apple polyphenol (AP), Hop bract polyphenol (HBP), EGCG, KYT-1 (Arg-gingipain inhibitor); and KYT-36 (Lys-gingipain inhibitor) in combination	In vitro	*P. gingivalis*	OSCC cells	Inhibition of protease activated receptor 2 (PAR2) and PAR4 mRNA expressions, pMMP-9 activation, and cellular invasion Reduced activation of heat shock protein 27 and Ets1 and nuclear translocation of nuclear factor-kappa B (NFκ-B)
Widén et al., 2015 [73]	Blackcurrant and sea buckthorn juices	In vitro	*S. mitis*, *S. mutans* *S. sanguinis*, *S. gordonii*, *S. aureus*, *S. epidermidis* and *P. aeruginosa.*	N/A	Inhibition of bacterial growth
Shahzad et al., 2015 [74]	Forty-eight purified (HPLC grade) Polyphenol compounds	In vitro	*S. mitis* *A. actinomycetemcomitans* *F. nucleatum* *P. gingivalis*	N/A	Antibacterial activities against periodontopathic bacteria in both planktonic and biofilm modes of growth
Kong et al., 2015 [77]	Theaflavins	In vitro	*P. gingivalis*	Human gingival fibroblasts (HGFs) from healthy gingival tissue.	Antimicrobial effects against both planktonic culture and biofilm of *P. gingivalis* Inhibition of the proteinase activities of *P. gingivalis* collagenase and gingipains Reduction in the secretion and mRNA expression of MMP-1 & MMP-2 by HGFs stimulated with *P. gingivalis*
Ben Lagha et al., 2015 [89]	Wild Blueberry (*Vaccinium angustifolium* Ait.) Polyphenols	In vitro	*F. nucleatum*	U937-3xκB cells	Inhibition of *F. nucleatum* growth and biofilm formation Inhibited the activation of NF-κB induced by *F. nucleatum* Inhibited the secretion of IL-1β, TNF-α, IL-6, MMP-8 & MMP-9
Tipton et al., 2014 [90]	Cranberry high molecular weight non-dialyzable material (NDM)	In vitro	N/A	Human gingival epithelial cells [Smulow-Glickman (S-G)]	Decreased nuclear levels of IL-1b-activated NF-jB (p65) & AP-1 (phospho-c-Jun), inhibited IL-6 production.
Jang et al., 2014 [78]	Baicalein	In vitro	*S. mitis* *S. mutans* *S. sanguinis* *S. sobrinus* *S. oralis* *Streptococcus ratti* *F. nucleatum* *A. actinomycetemcomitans* *P. gingivalis*	N/A	Inhibition of bacterial growth
Tipton et al., 2013 [91]	Cranberry high molecular weight non-dialyzable material (NDM)	In vitro	N/A	Human gingival epithelial cells and human gingival fibroblasts	Inhibition of constitutive and IL-17-stimulated IL6 & IL-8 production by epithelial cell and gingival fibroblasts
Zdarilová et al., 2010 [82]	Polyphenolic fraction of *L. caerulea berries*	In vitro	*P. gingivalis*	Human gingival fibroblasts from healthy donors free of periodontal disease.	Reduction of ROS production, intracellular glutathione (GSH) depletion, and lipid peroxidation Inhibited LPS-induced up-regulation of IL-1β, IL-6 and TNF-α Suppressed expression of cyclooxygenase-2 (COX-2)

### 3.3. Polyphenols and Halitosis

Halitosis, commonly known as bad breath, is a dental condition characterized by an unpleasant and offensive odor emanating from the oral cavity. Halitosis is highly prevalent, including physiological or pathological halitosis. The latter can be classified further into oral or extraoral halitosis, depending on the source of the odor [102]. Pathological halitosis is mainly caused by the presence of Gram-negative bacteria in the oral cavity that produces volatile sulfur compounds (VSC) such as hydrogen sulfide (H_2_S), methyl mercaptan (CH_3_SH), and dimethyl sulfide (C_2_H_6_S) [103,104]. Other factors contributing to halitosis include poor oral hygiene, tongue biofilm, food impacts, gum diseases, dental abscesses, dry mouth, oral ulcers, respiratory infections, and other oral or systemic conditions [102,105]. Treatments for halitosis include the improvement of oral hygiene, utilization of antibacterial mouthwashes, and medicinal plants with antimicrobial properties.

Polyphenolic compounds have been found to play an important role in the treatment of halitosis (Table 4). These compounds, which are naturally present in fruits, vegetables, and medicinal plants, possess antimicrobial properties and can help inhibit the bacteria that contribute to the unpleasant odor [103]. Studies have shown that polyphenol-rich plant extracts, such as those from pomegranate, cinnamon, rosemary, and other medicinal plants, exhibit antibacterial activity against oral microorganisms, including those involved in VSC production [103,106]. These extracts have been found to inhibit the growth of bacteria in the oral cavity and reduce the formation of VSC, addressing the root cause of halitosis. Liu et al. found that thinned-young apple polyphenols (YAP) exerted antibacterial effects by destroying the cell membrane of halitosis-related bacteria, including *P. gingivalis*, *P. intermedius*, and *F. nucleatum* [107].

The exact mechanisms by which polyphenols exert their antibacterial and anti-halitosis effects are still being investigated. However, their antioxidant properties are believed to play a role. Polyphenols can neutralize harmful free radicals and oxidative stress, which are known to contribute to oral health problems, including halitosis (Figure 2, Table 4) [103,108]. Additionally, these compounds may interfere with bacterial metabolism and disrupt the formation of VSC. Morin et al. reported that green tea extract and its major constituent EGCG inhibited the growth of *S. moorei* by eradicating its pre-formed biofilms. Furthermore, the activity of β-galactosidase in *S. moorei*, which is crucial for VSC production, was inhibited in a dose-dependent manner by EGCG in green tea [106]. According to the findings of a double-blinded and placebo-controlled clinical trial, the use of green tea mouthwash (green tea extract contains more than 80% of total catechins) for 4 weeks resulted in a significant reduction in VSC levels in individuals with gingivitis [109]. Lodhia et al. demonstrated that green tea powder significantly reduced the concentration of both H_2_S and CH_3_SH gases in subjects after administration [110]. Polyphenol-rich extracts from medicinal plants as an alternative treatment for halitosis offer a natural and safer approach compared to conventional mouthwashes that may have potential side effects. However, further research is needed to determine the optimal concentrations and formulations of these extracts for halitosis treatment, as well as their long-term effects. Incorporating polyphenols into oral care products or dietary supplements may provide a natural and effective solution for individuals suffering from bad breath.

**Table 4:** Effects of polyphenols on halitosis.

Study Group	Active Components	Study Design	Pathogens	Cells/Tissues	Results
Liu. et al., 2021 [107]	Thinned young apple polyphenols (YAP)	in vitro	*P. gingivalis*, *P. intermedius*, *F. nucleatum*	N/A	Inhibition of halitosis-related bacteria growth Destroyed integrity and permeability of the cell membrane
Veloso et al., 2020 [103]	Crude extracts obtained from Jucá, Cinnamon, Mallow, Pomegranate, Rosemary, Macassá, Clove, and Tamarind	in vitro	*P. gingivalis*, *P. intermedia*, *F. nucleatum*, *P. micra*	N/A	Pomegranate extract was the only extract that inhibited all the evaluated microorganisms
Morin et al., 2015 [106]	EGCG from green tea	in vitro	*S. moorei*	N/A	Inhibited *S. moorei* growth and bacterial adherence Reduction of the biofilm formation Suppression of bacterial β-galactosidase activity
Rassameemasmaung et al., 2008 [109]	Green tea mouthwash	Double-blinded and placebo-controlled clinical trial in 60 gingivitis patients	N/A	N/A	Reduced VSC level in gingivitis subjects after rinsing for 4 weeks
Lodhia et al., 2008 [110]	Green tea powder	In vitro and in vivo studies; Analyze the concentration of both H2S and CH3SH gases	N/A	N/A	Green tea exhibited significant temporary Reduced oral malodor due to its disinfectant and deodorizing properties

### 3.4. Polyphenols and Oral Cancer

Oral cancers, particularly OSCCs, account for approximately 90% of head and neck cancers [111]. OSCCs are characterized by the development of malignant cells in the squamous cells that line the oral cavity, including the lips, tongue, gums, lining of the cheeks, and floor of the mouth. The progression of OSCCs typically starts with changes in the normal oral mucosa, leading to the formation of precancerous lesions such as leukoplakia or erythroplakia [112]. The development of these tumors is influenced by multiple factors, starting with the cancerous lesions of the normal mucosa and eventually progressing to metastasis. Among the main risk factors, excessive alcohol consumption and tobacco use are considered leading causes due to their proinflammatory effects [113]. Despite advancements in therapeutic strategies such as chemotherapy, radiotherapy, and surgery, the 5-year survival rate of OSCCs was less than 50% [114]. Therefore, novel relief agents and treatment approaches for OSCCs are a critical need.

In the past decade, the potential preventive and therapeutic effects of various polyphenols against OSCCs have been reported. Polyphenols interfere with the cell cycle and inhibit the invasion of cancer cells into other tissues and organs [12]. They have been shown to have anti-inflammatory, antioxidant, and anti-carcinogenic effects as well [7] (Figure 2, Table 5).

Polyphenolic compounds found in green and black tea, such as catechins, theaflavins, polymeric thearubigins, and EGCG, suppress the growth and division of oral cancer cells in vitro [49,115,116,117]. Studies on hamsters and rats also showed similar effects. Administration of black tea polyphenols significantly decreased tumor incidence in the buccal pouch of male Syrian hamsters [118]. Srinivasan et al. demonstrated that the treatment of green tea polyphenols declined the number of tumors, tumor volume, and OSCCs in 4-nitroquinoline-1-oxide -induced rats [119]. Sharma et al. reported that the polyphenolic flavonoids found in defatted seeds of *Azadirachta indica* and *Momordica charantia* exhibited antiproliferative activity against Human oral epidermal carcinoma KB cells [120]. A study by King et al. showed the dose-dependent inhibition of cellular proliferation in OSCC cells with grape seed PAC. In addition, the administration of PAC reduced the increased proliferation of OSCCs after transfection with HPV 16 (human papillomavirus 16) [121].

The effect of polyphenols on apoptosis in oral cancer cells has also been studied (Table 5). Treatment of OSCC cells with cranberry and grape seed extracts partially up-regulated the expression of apoptosis-specific molecules, such as caspase-2 and caspase-8 [116]. Specifically, treatment with PAC ranging from 50 to 70 μg/mL for OSCCs was found to increase caspase-2 and caspase-8 expression [122]. Black tea polyphenols were also found to reduce the incidence of DMBA (7,12-dimethylbenz [a]anthracene)-induced hamster buccal pouch carcinogenesis by modulating biomarkers of cell proliferation, angiogenesis, and apoptosis [118]. These results demonstrate that polyphenol-associated apoptosis is mitochondria-targeted and caspase 8 dependent.

Polyphenols interfere with the signaling pathways involved in oral cancer development and progression (Table 5). Fan et al. illustrated that anthocyanins from a species of black rice have the potential to inhibit the metastasis of CAL 27 cells. This nutrient reduces the expression of MMP-2, MMP-9, and NF-κB p65 through the suppression of the PI3K/Akt pathway and the inhibition of NF-κB [123]. A study found that EGCG demonstrated a dose-dependent inhibitory effect on the invasion and migration of OC2 cells without causing cytotoxicity (ref). Additionally, EGCG was found to decrease the expression levels of MMP-2, MMP-9, and u-PA in a concentration-dependent manner [124]. Moreover, it significantly inhibits the invasion, motility, migration, and secretion of MMP-2 and u-PA in SCC-9 oral cancer cells by attenuating p-FAK and p-Src. EGCG also inhibited the tumor growth of SCC-9 cells in vivo via cancer cell xenografted nude mice model [125]. These findings suggest that polyphenols have the potential to inhibit the invasion and migration of oral cancer cells through the regulation of key proteins involved in these processes (Figure 2).

Evidence from human studies regarding the function of polyphenols has also accumulated. Recently, a randomized placebo-controlled phase 1 trial of APG-157 (a botanical drug containing multiple polyphenols, including curcumin) in oral cancer suggested that the material can be easily absorbed, showing an inhibitory effect on cytokines and tumor-associated microbes [126]. APG-157 reduced the levels of IL-1β, IL-6, and IL-8 in the saliva of patients, and the analysis of microbial flora showed a decrease in *Bacteroides* species [126]. These results illustrated that polyphenols are chemopreventive and are potential therapeutic agents against OSCCs. However, the bioavailability and handling of polyphenols can be challenging due to their unfavorable physicochemical properties. Further research is needed to develop effective drug delivery systems that can improve the clinical use of polyphenols in the treatment of OSCCs.

**Table 5:** Effects of polyphenols on Oral cancers.

Study Group	Active Components	Study Design	Cells/Tissues/Animals	Results
Sharma et al., 2023 [120]	Defatted seeds of *Azadirachta indica* and *Momordica charantia*	in vitro	Human oral epidermal carcinoma KB cell line	Bioactive extracts had antiproliferative activity and antioxidant capacity Suppressed KB cells Binding efficacy against tumor suppressor gene regulatory function
Nimbalkar et al., 2022 [127]	Polymeric black tea polyphenols (PBPs)/thearubigins (TRs)	in vivo	Hamster model of oral carcinogenesis	Modulated EGFR pathway associated with cell proliferation, hypoxia, and angiogenesis.
Liu et al., 2022 [128]	Grape seed proanthocyanidins (PAC)	in vitro	Oral squamous cell carcinoma cell lines SCC-4, Human oral squamous cell carcinoma cell lines HSC-3	Developed a complex coacervates-based delivery of PAC Inhibited cell proliferation, migration, and invasion of cancer cells Reduction of MMP-2, MMP-9, and MMP-13 Suppressed protein kinase B (Akt) pathway
Basak et al., 2020 [126]	APG-157 (a botanical drug containing multiple polyphenols, including curcumin)	Phase I clinical trial (*n* = 25)	N/A	Reduced IL-1β, IL-6, and IL-8 concentrations in the salivary supernatant fluid of patients with cancer Reduction in Bacteroidetes species in cancer subjects Up-regulation of genes associated with differentiation and T-cell recruitment to the tumor microenvironment.
Sheng et al., 2018 [129]	Resveratrol, epigallocatechin gallate (EGCG), and tannic acid	in vitro	Normal human oral keratinocytes NHOKs, Human oral squamous cell carcinoma cell lines HSC-2	Resveratrol in combination with doxorubicin additively augmented doxorubicin cytotoxicity in both types of cells. EGCG and tannic acid alleviated the toxicity caused by doxorubicin in keratinocytes, primarily by reducing doxorubicin-induced necrosis in normal human oral keratinocytes
Huang et al., 2018 [130]	Hydrogels formed ellagic acid (EA) and EGCG	in vitro	Human oral cancer cell line CAL-27	Long-term steady-state release of bioactive EA Reduced viability of CAL-27 human oral cancer cells
Fan et al., 2015 [123]	Anthocyanins from black rice (*Oryza sativa* L.)	in vitro	Human oral cancer cell line CAL-27	Suppression of CAL 27 cell metastasis Reduction in MMP-2, MMP-9, and NF-κB p65 expression through the suppression of PI3K/Akt pathway Inhibition of NF-κB levels
Chang et al., 2012 [115]	Black tea polyphenol extracts (BTE)	in vitro and in vivo	Oral squamous cell carcinoma cell lines SCC-4 5-week-old immunodeficient nude mice	Up-regulation of epithelial markers such as E-cadherin Inhibition of mesenchymal markers such as snail-1 and vimentin Inhibition of the tumor growth of SCC-4 cells via cancer cell xenografted nude mice mode
Chen et al., 2011 [125]	Green tea polyphenol epigallocatechin-3 gallate (EGCG)	in vitro and in vivo	Oral squamous cell carcinoma cell lines SCC-9 5-week-old immunodeficient nude mice	Inhibition of p-focal adhesion kinase (p-FAK), p-Src, snail-1, and vimentin Inhibition on the tumor growth of SCC-9 cells in vivo
Chatelain et al., 2011 [116]	Cranberry and grape seed extracts	in vitro	Oral squamous cell carcinoma cell lines CAL-27 and SCC-25	Inhibition of oral cancer proliferation Up-regulation of caspase-2 and caspase-8 levels
Kingsley et al., 2010 [122]	Proanthocyanidins (PAC)	in vitro	Oral squamous cell carcinoma cell lines CAL-27 and SCC-25	Inhibition of oral cancer proliferation Up-regulation of caspase-2 and caspase-8 levels Down-regulation of specific cell-cycle regulators
Srinivasan et al., 2008 [119]	Green tea polyphenols	in vivo	Wistar strain male albino rats	Reduced the number of tumors, tumor volume, and oral squamous cell carcinoma
Letchoumy et al., 2008 [131]	Black tea polyphenols Polyphenon-B and BTF-35	in vivo	Male Syrian hamsters aged 6–10 weeks weighing between 90–110 g	Decreased tumor incidence, oxidative DNA damage, phase I enzyme activities Reduction in CYP1A1 and CYP1B1 Enhanced phase II enzyme activities in the buccal pouch and liver
Mohan et al., 2007 [117]	Green and black tea polyphenols alone and in combination with bovine milk lactoferrin (bLF)	in vitro	Human tongue squamous carcinoma CAL-27 and normal human gingival fibroblast (HGF) cells	Inhibition of CAL-27 cell growth Transduced the apoptosis signal via the generation of reactive oxygen species and decrease in the Bcl-2/Bax ratio Activation of caspase-3
Letchoumy et al., 2007 [118]	Black tea polyphenols, Polyphenon-B, and BTF-35	in vivo	Male Syrian hamsters aged 6–10 weeks weighing between 90–110 g	Reduced the incidence of DMBA-induced hamster buccal pouch carcinomas by modulating markers of cell proliferation, cell survival, tumor infiltration, angiogenesis, and apoptosis
King et al., 2007 [121]	Proanthocyanidin (PAC)	in vitro	Human oral squamous cell carcinoma CAL 27, human cervical carcinoma Ca Ski, human cervical adenocarcinoma GH354, and human foreskin fibroblasts Hs27 cell lines	Suppression of cellular proliferation of OSCC Induced apoptosis in cervical and oral cancer cell lines
Ho et al., 2007 [124]	Epigallocatechin-3-gallate (EGCG)	in vitro	OC2 cells	Inhibited invasion and migration of OC2 cells Decreased expressions of MMP-2, MMP-9, and uPA in a dose-dependent manner

## 4. Discussion

**Figure 3:**
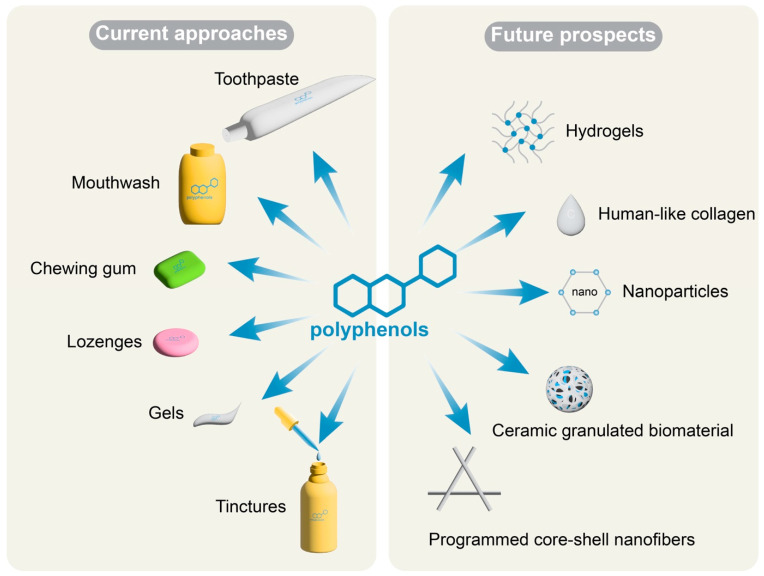
Current approaches and prospects of polyphenols-based oral hygiene. Diverse strategies are employed in polyphenols-based oral hygiene, ranging from conventional methods to cutting-edge developments in biomaterials. Current approaches include widely-used products for routine cleaning, e.g., toothpaste and mouthwash; saliva stimulation, e.g., chewing gum and lozenges; or even polyphenols-containing gels and tinctures. Emerging trends include the development of hydrogels, human-like collagen, and other new biomaterials, such as nanoparticles, programmed core-shell nanofibers, and ceramic granulated biomaterials.

Polyphenols have gained significant attention in biomedical fields due to their various beneficial properties, including antioxidant ability, antibacterial activity, and anti-inflammatory activity. Moreover, numerous researches have shown that polyphenols play critical roles in maintaining oral health and oral microbiota, which benefits oral health and overall well-being for humans. However, it is important to note that the therapeutic activities of polyphenols are limited by their poor bioavailability. Polyphenols are typically applied in the form of mouthwashes, gels, or tinctures in oral infections, such as gel containing *Scutellaria baicalensis*(*S. baicalensis*) root extract (commercially known as Baikadent^®^) widely used in the treatment of oral inflammation (Figure 3). The direct contact of functional molecules with pathogens on the mucosa or teeth surface allows the antibacterial effects. However, the penetration of polyphenols into deeper regions of the oral mucosa may be limited, depending on the mucosal surface type and the duration of action. Additionally, the poor stability of certain polyphenols, such as EGCG from tea, further limits their potential application.

Further research is needed to explore the bioaccessibility of polyphenols and develop modern delivery systems to enhance their effectiveness. In addition, the stability and bioavailability of polyphenols can be improved through techniques such as nanoencapsulation, which protects sensitive ingredients from degradation and enhances their absorption across biological barriers. Recently, several studies have demonstrated the potential applications of polyphenols combined with different materials in the treatment of oral diseases (Figure 3). He et al. developed a programmed core-shell nanofiber that allows the sequential and controlled release of tea polyphenols and AdipoRon. The nanofibers can alleviate periodontal tissue inflammation and promote bone regeneration to repair periodontitis-related alveolar bone defects in vivo. These findings suggest that the programmed core-shell nanofibers have potential applications in the treatment of periodontitis [97]. A ceramic granulated biomaterial containing phenolic molecules was considered an innovative regenerative approach in periodontal therapy [80]. Nanovesicles in-situ gel based on the EGCG phospholipid complex improved its stability and bioavailability [61]. Polyphenols-linked hydrogels are also new directions in the treatment of oral diseases for the gradual release of bioactive components [132,133]. Ellagic acid (EA)-linked hydrogels were found to provide long-term sustained release of bioactive polyphenols and significantly suppress the CAL-27 human oral cancer cell viability [130]. Using the oxidative self-polymerization principle, Qi et al. prepared polyphonic nanoparticles, which were encapsulated into thermosensitive type in-situ hydrogel. The effective constituent can be released continuously for 96 h under the periodontitis environment [96]. It has been demonstrated that human-like collagen has properties that contribute to tissue healing and regeneration, including promoting collagen deposition, regulating growth factors, and reducing inflammation [134,135]. Due to its outstanding biocompatibility, minimal immunogenicity, and ability of natural degradation polyphenols combined with human-like collagen have great potential in the treatment of oral diseases.

Polyphenols have a stimulating effect on host cells. Several studies suggested that polyphenols, such as EGCG, theaflavin, and resveratrol, can induce human beta-defensin (hBD) secretion in oral epithelial cells [136,137,138]. Lombardo et al. found that tea polyphenols induced hBD secretion in gingival epithelial cells and protected hBDs from proteolytic degradation by *P. gingivalis* [136]. When oral pathogens invade the oral tissues or biofilms form on the tooth surface, antimicrobial peptides are released to combat the pathogens and maintain microbial balance [137]. In addition to their direct antimicrobial effects, antimicrobial peptides also have other beneficial functions in oral health, such as modulating the inflammatory response and promoting wound healing and tissue repair in the oral cavity [138]. Polyphenol-induced antimicrobial peptides in the oral cavity regulate the oral microbiota and play a role in oral health. The interaction between polyphenol, pathogen, and host cells opens up a new research direction in the future.

The potential benefits of polyphenols in managing and treating oral diseases suggest that these molecules should be carefully evaluated in clinical settings. While there are many studies conducted in laboratory and animal models that demonstrate the potential benefits of polyphenols in oral health, strong evidence from well-designed clinical trials is still limited. Future research should focus on the applications in humans and evaluate the long-term benefits and safety of polyphenols in preventing and treating oral diseases. With the advances in precision medicine, there is a growing interest in personalized treatment approaches. Polyphenols, with their diverse pharmacological activities, could be tailored to individuals based on their specific oral health needs and microbial profiles. This personalized approach could lead to more targeted and effective oral health interventions.

While dietary polyphenols offer numerous benefits, they are not a panacea but rather should be part of a balanced diet. Various foods contain different types and amounts of polyphenols, and incorporating a variety of fruits, vegetables, nuts, and grains into your diet can maximize the benefits of dietary polyphenols. Additionally, the intake of dietary polyphenols should be moderate to avoid potential adverse effects, as excessive consumption may not always be beneficial and may even lead to digestive issues such as stomach upset, diarrhea, or constipation. Additionally, some individuals may be sensitive or allergic to specific polyphenols, resulting in adverse reactions such as skin rashes or headaches. Moreover, polyphenols can interact with medications, potentially reducing their effectiveness or causing unwanted side effects. Furthermore, utilizing polyphenolic lozenges, toothpaste, mouthwash, and other products to maintain good oral hygiene practices helps remove plaque, balance the oral microbiota, and prevent tooth decay and other oral disease.

It should be emphasized that tooth decay, gum disease, and bad breath often occur during aging and further exacerbate our health condition. Polyphenols possessing strong antioxidant properties can help combat oxidative stress, which is particularly important for aging. By reducing cellular damage and inflammation, polyphenols may function to slow the aging process. Several polyphenols such as curcumin, resveratrol, quercetin, EGCG, and tyrosol, were found to have anti-aging effects and could promote oral health by combating oxidative stress and inflammation [139]. Therefore, polyphenols as functional foods might not only serve as a novel strategy to promote oral health but also prevent oral disease indirectly through their anti-aging effects.

## 5. Conclusions

Oral and periodontal diseases are primarily caused by an imbalance in the oral microbiome and the subsequent host immune response [30]. The studies summarized in this review demonstrated the antimicrobial properties of the polyphenols, which help in inhibiting the growth of pathogenic microorganisms and maintaining a balanced oral microbiota. Polyphenols also have antioxidant and anti-inflammatory activities, reducing inflammation and oxidative stress in the oral cavity [6]. Additionally, polyphenols can inhibit the attachment and biofilm formation of bacteria, preventing dental plaque formation and promoting oral hygiene [140]. Overall, polyphenols are crucial for the maintenance of oral health and the balance of the oral ecosystem, which is vital for overall human health. Future studies should aim to reveal more information about the interaction between polyphenols, pathogens, and host cells. Long-term clinical studies of reliable polyphenol delivery systems and combination therapy with antibiotics will also be useful.

In summary, research on the functions, working mechanisms, and applications of food-originated polyphenols in oral health is important for the overall well-being of humans. The progressions in polyphenol-based microecology control and oral disease prevention highlight new directions for safe, convenient oral health maintenance. More importantly, the exploration of polyphenols opens up new avenues for the management of oral disease, and the achievements in animal studies and clinical trials provide insight to advance treatments and cost-effective healthcare strategies in the future.

## Figures and Tables

**Table 1:** Classification and chemical structure of major classes of dietary polyphenols.

Polyphenols	Subclasses and Examples	Food Sources
*Lignans*	e.g., secoisolariciresinol, matairesinol 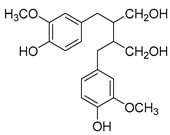 (Secoisolariciresino)	Linseed, lentils, garlic, asparagus, carrots, pears, and prunes.
*Stibenes*	e.g., resveratrol, trans-resveratrol 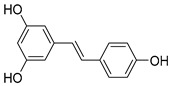 (Resveratrol)	Grapes, pomegranates, and groundnuts.
*Phenolic acids*	(A) Hydrobenzoic acids: protocatechuic acid, gallic acid, p-hydroxybenzoic acid 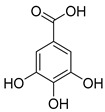 (gallic acid)	Blackberries, raspberries, strawberries, grapes, and black currants.
	(B) Hydroxycinic acids: caffeic acid, chlorogenic acid, coumaric acid, ferulic acid, sinapic acid 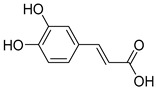 (caffeic acid)	Blueberries, kiwis, cherries, plums, apples, pears, peaches, chicories, artichokes, potatoes, and coffee.
*Flavonoids*	Anthocyanins: malvidin, cyanidin, pelargonidin, peonidin, delphinidin 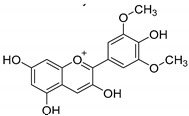 (malvidin)	Red, blue, and purple berries, red and purple grapes, aubergines, red cabbages, rhubarbs, red wine, and cherries.
	Flavonols: quercitin, kaempferol, mycricetin 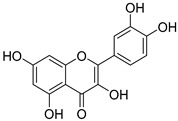 (quercitin)	Leeks, gingers, broccoli, red cabbages, yellow onions, cherries, tomatoes, blueberries, apricots, apples, black grapes, and teas.
	Flavanones: hesperidin, naringenin, erioclictyol 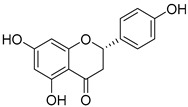 (Naringenin)	Citrus fruits (oranges, grapes, and lemons) and their juices.
	Flavones: apigenin, luteolin 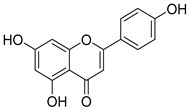 (Apigenin)	Oregano, celery, thyme, parsley, capsicums, and pepper.
	Isoflavonones: daidzein, genistein, glycitein 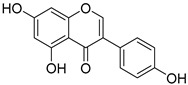 (Genistein)	Milk, tofu, soybeans, tempeh, miso, and legumes.
	Monomeric flavanols: catechin, epicatechin, epigallocatechin, epigallocatechin gallate Polymeric flavanols: theaflavins, thearubigins 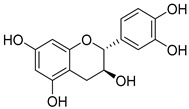 (catechin)	Grapes, chocolate, red wine cocoa, berries, apples, apricots, black beans, green and black teas.
	Proanthocyanidins 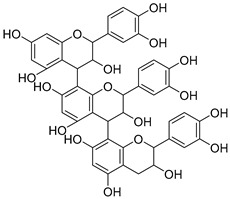 (Proanthocyanidins)	Chocolate, apples, berries, red grapes, and red wine.

## Data Availability

Not applicable.
